# Jottings

**Published:** 1910-02-26

**Authors:** 


### Jottings.

The King has sent gifts of pheasants to the London Throat Hospital, Great Portland Street.

The annual meeting of the governors of the Prince of Wales' General Hospital, Tottenham, has been deferred until April, when H.R.H. the Princess Louise, the President, will preside.

The annual court of the Corporation of King's College Hospital was held in the Board-room on Thursday, February 24, at half-past four o'clock, when the annual report of the hospital was presented and adopted.

At the annual general meeting of the Governors of the Royal Westminster Ophthalmic Hospital, held at the hospital on Thursday, February 24, the annual report was read and adopted, and the hospital officers re-elected for the ensuing year.

The building at the Hospital for Women, Soho Square, being now more than 50 years old, was found to be insanitary, inadequate, and unsuitable, and has therefore been rebuilt in conformity with the plan approved by King Edward's Hospital Fund. About £15,000 will be required, and the balance is to be obtained by a bazaar in the new buildings, under Royal and distinguished patronage.

The Queen, Princess Henry of Battenberg, the Duchess of Albany, and the Duchess of Teck have given their patronage to the special *matinée* at His Majesty's Theatre on May 23 in aid of the Royal Waterloo Hospital for Children and Women. The *matinée* is under the direction of Princess Alexander of Teck, with a representative committee, and Mrs. Alfred Mond represents the hospital.

The Princess of Wales will open the new building of the Hospital for Invalid Gentlewomen, 19 Lisson Grove, N.W., on Monday, March 7, at 3.30. The hospital can be inspected on March 8 and 9 from 2 till 5 p.m.

The Fulham Guardians Infirmary Committee has decided that, subject to the sanction of the Local Government Board, a second assistant medical officer be appointed, in addition to the present staff, at a salary of £110 per annum, increasing £15 annually to a maximum of £140 per annum, in addition to allowances.

The annual festival dinner of the Royal Hospital for Incurables, Putney Heath, will be held at the Grocers' Hall. Princes Street, E.C., on Wednesday, May 11, at 6.30 p.m., for 7 o'clock precisely. Ladies are cordially invited, and gentlemen who are willing to act as stewards should kindly communicate with the Secretary, Mr. Charles Cutting.

The Annual Court of Governors of East London Hospital for Children was held at the hospital on Monday, February 21, at 4 p.m., when the annual report and balance sheet were submitted, the general committee, the board of management, and honorary officers elected, and other business transacted.

The annual report of the East London Hospital for Children, Shadwell, referred to the regrettable deficit of nearly £700, and stated that the falling-off in annual subscriptions was the worst feature; and, further, that plans were before King Edward's Hospital Fund for the extension of the out-patient department at a cost of £4,000, which it was hoped would be approved.

At the annual court of governors of the Royal Berkshire Hospital, which was held at Reading last Tuesday, a demand made by the Reading Trades and Labour Council that in all contracts given out by the hospital for work a "fair wage clause" should be inserted was agreed to, and the claim asserted by the Home Office to inspect the laundry under the Factory Acts was upheld in a friendly suit before the magistrates.

At the annual meeting of the governors of Charing Cross Hospital, last week, the report of the council stated that during the year the income had just sufficed to meet the ordinary expenses and to provide the annual payments (£4,925) for interest and sinking fund in connection with the mortgage debt of £85,000. The ordinary income was £17,998, and the ordinary expenditure was £17,670.

Sir Savile Crossley presided at the first meeting of the reconstituted board of delegates of the Hospital Saturday Fund Association for 1910, held last Saturday at the central Offices, Gray's Inn Road. Sir Savile Crossley was re-elected Chairman of the Association, and Mr. W. F. Chalkley, Chairman of the Council. The Hon. H. L. W. Lawson, M.P., was re-elected Hon. Treasurer. The annual "Hospital Saturday" collection was fixed for October 15.

The new Melbourne General Hospital, commenced early this year, is to be the largest in Australia on the pavilion system. The plans accepted by the committee present a line of five buildings united by covered ways. The central block, of five stories, is flanked by two buildings of six stories, and these again are connected with the outpatients' department (four stories) on the west and the pathological block of similar height on the east. A detached building further east is to serve as a refractory ward. The total number of beds will be 400.

